# Nopal (*Opuntia ficus indica*) protects from metabolic endotoxemia by modifying gut microbiota in obese rats fed high fat/sucrose diet

**DOI:** 10.1038/s41598-017-05096-4

**Published:** 2017-07-05

**Authors:** Mónica Sánchez-Tapia, Miriam Aguilar-López, Claudia Pérez-Cruz, Edgar Pichardo-Ontiveros, Mei Wang, Sharon M. Donovan, Armando R. Tovar, Nimbe Torres

**Affiliations:** 1 Departamento de Fisiología de la Nutrición, Instituto Nacional de Ciencias Médicas y Nutrición Salvador Zubirán, Vasco de Quiroga No 15, Ciudad de Mexico, 14080 Mexico City, Mexico; 2 Departamento de Farmacología, Laboratorio de Neuroplasticidad y Neurodegeneración, CINVESTAV, Ciudad de Mexico, 07360 Mexico City, Mexico; 3 Department of Food Science and Human Nutrition, University of Illinois, Illinois, IL 61801 USA

## Abstract

Current efforts are directed to reducing the gut dysbiosis and inflammation produced by obesity. The purpose of this study was to investigate whether consuming nopal, a vegetable rich in dietary fibre, vitamin C, and polyphenols can reduce the metabolic consequences of obesity by modifying the gut microbiota and preventing metabolic endotoxemia in rats fed a high fat and sucrose diet. With this aim, rats were fed a high fat diet with 5% sucrose in the drinking water (HFS) for 7 months and then were fed for 1 month with HFS + 5% nopal (HFS + N). The composition of gut microbiota was assessed by sequencing the 16S rRNA gene. Nopal modified gut microbiota and increased intestinal occludin-1 in the HFS + N group. This was associated with a decrease in metabolic endotoxemia, glucose insulinotropic peptide, glucose intolerance, lipogenesis, and metabolic inflexibility. These changes were accompanied by reduced hepatic steatosis and oxidative stress in adipose tissue and brain, and improved cognitive function, associated with an increase in *B*. *fragilis*. This study supports the use of nopal as a functional food and prebiotic for its ability to modify gut microbiota and to reduce metabolic endotoxemia and other obesity-related biochemical abnormalities.

## Introduction

Increased consumption of foods high in fat and sugar are the main contributors of obesity^1^, gut microbiota dysbiosis^2^, inflammation^3^, gut barrier disruption^4^ and cognitive decline^5^. Evidence shows a causal link between the intestinal microbiota and insulin resistance through different mechanisms including the increase in the production of short chain fatty acids by increasing hepatic lipogenesis^6^, the conversion of primary to secondary bile acids by the gut microbiota regulating the membrane-type receptor for bile acids TGR5^7^, and by a chronic low-grade inflammation mediated by lipopolysaccharide (LPS) activating a cell signalling pathway that induces an inflammatory response and cytokine secretion. Particularly, the activation of TLR4 by LPS downregulates the insulin signalling pathway inducing insulin resistance^8^. Insulin regulates the activity of specific brain areas that are important for memory and the regulation of whole-body metabolism. It has been established that insulin resistance in the brain is associated with deficits in cognitive flexibility^9^. Efforts to improve insulin sensitivity have been directed to reducing dysbiosis of the gut microbiota, with one of the most effective strategies being modification of the diet to reduce insulin resistance and increase systemic and local intestinal anti-inflammatory activities^10^. We have demonstrated that the pads or cladodes of nopal (*Opuntia ficus indica*), a vegetable extensively consumed in Mexico and southern parts of the United States, are rich in dietary fiber and bioactive compounds with antioxidant activity^11^, such as flavonoids, flavonols, carotenes and ascorbic acid. Nopal is also low in calories (27 kcal/100 g) and has low glycemic and insulinemic indexes^12^. In subjects with type 2 diabetes, nopal has an antihyperglycemic effect accompanied by a low stimulation of glucose insulinotropic peptide (GIP) secretion^12^. Previous studies have demonstrated that obese Zucker fa/fa rats fed 7 weeks with nopal decreased hepatic steatosis by increasing fatty acid oxidation and decreasing oxidative stress. We demonstrated that consumption of nopal also increases the phosphorylation of IRS-1 and AKT in the liver, indicating an increase in insulin signaling^13^. However, the underlying mechanisms for the possible beneficial effects of nopal remain largely unknown. Here, we investigate whether obese rats fed with nopal can modify gut microbiota to attenuate metabolic endotoxemia and the subsequent insulin resistance, biochemical abnormalities and improve cognitive function in rats fed a sucrose-enriched high fat diet.

## Results

### Nopal consumption attenuates body weight gain and modifies biochemical parameters in rats fed a high fat-high sucrose diet

**Figure 1:**
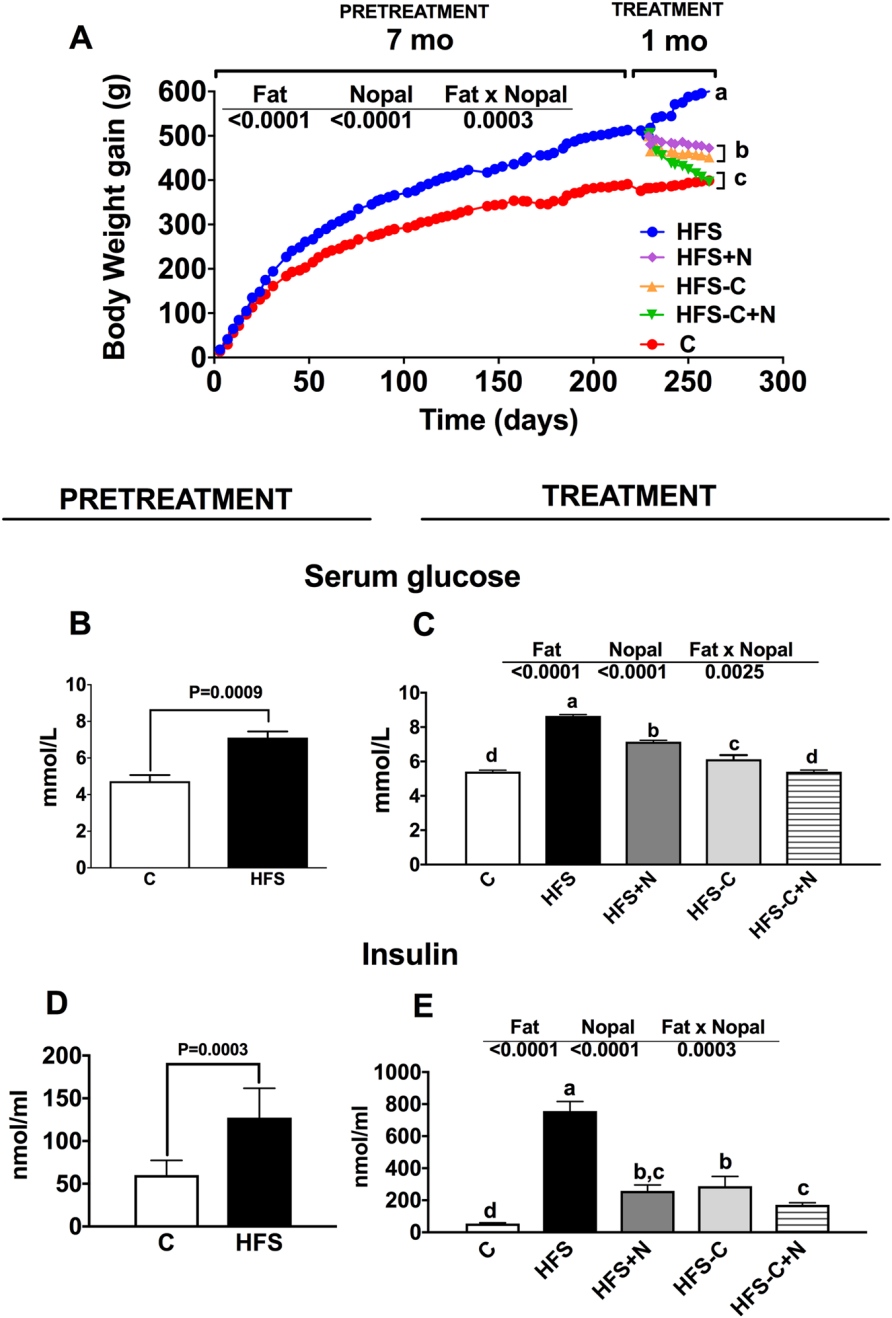
Effect of nopal on body weight gain, serum glucose and insulin concentrations. (**A**) Body weight gain of rats fed control diet (**C**) or high fat diet +5% sucrose in drinking water (HFS) during 7 months (pretreatment period). After this period, obese rats were switched to one of the experimental diets for 1 month (treatment period): HFS, HFS + N, HFS-C, and HFS-C + N. (**B**) Fasting serum glucose and (**D**) insulin after the pretreatment and (**C**), (**E**) treatment periods respectively. Values are shown as means ± SEM, n = 5 rats per group. Statistical analysis of the pretreatment period was assessed by student t-test. Comparisons among groups after the treatment period were analyzed by two-way ANOVA followed by Fisher’s post-hoc test. Different letters indicate significant differences among groups, a > b > c > d, P < 0.05. Comparisons between 2 groups were analyzed by t-student test.

**Figure 2:**
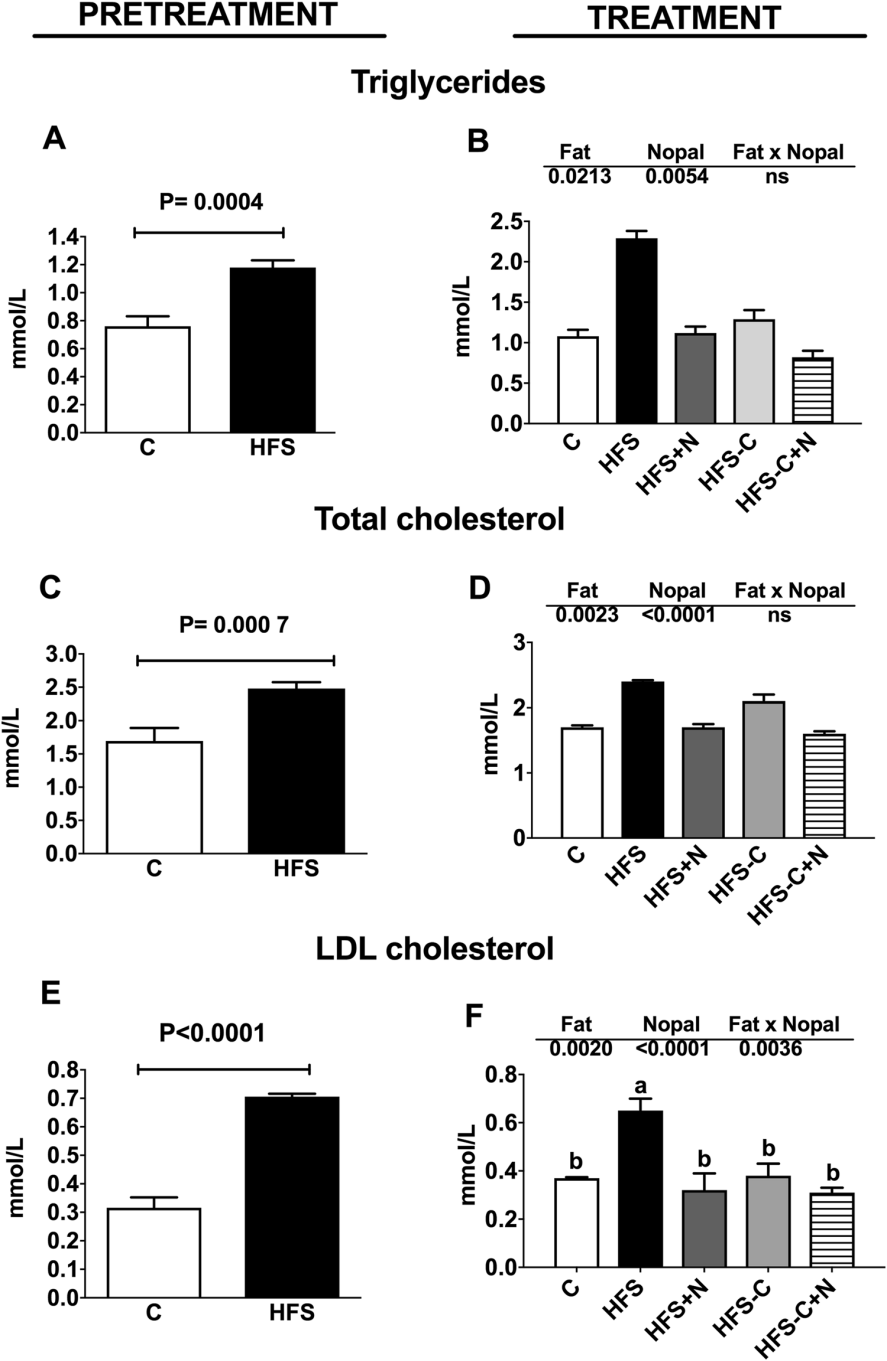
Effect of nopal consumption on blood lipids. (**A**) Fasting serum triglycerides, (**C**) total cholesterol and (**E**) LDL cholesterol of rats fed control diet (**C**) or high fat diet +5% sucrose in drinking water (HFS) during 7 months (pretreatment period). After this period, obese rats were switched to one of the experimental diets for 1 month (treatment period): HFS, HFS + N, HFS-C, HFS-C + N; (**B**), (**D** and **F**). Values are shown as means ± SEM, n = 5. Statistical analysis of the pretreatment period was assessed by student t-test. Comparisons among groups after the treatment period were analyzed by two-way ANOVA followed by Fisher´s post-hoc test. Different letters indicate significant differences among groups, a > b > c > d, P < 0.05. Comparisons between 2 groups were analyzed by student t test.

**Figure 3:**
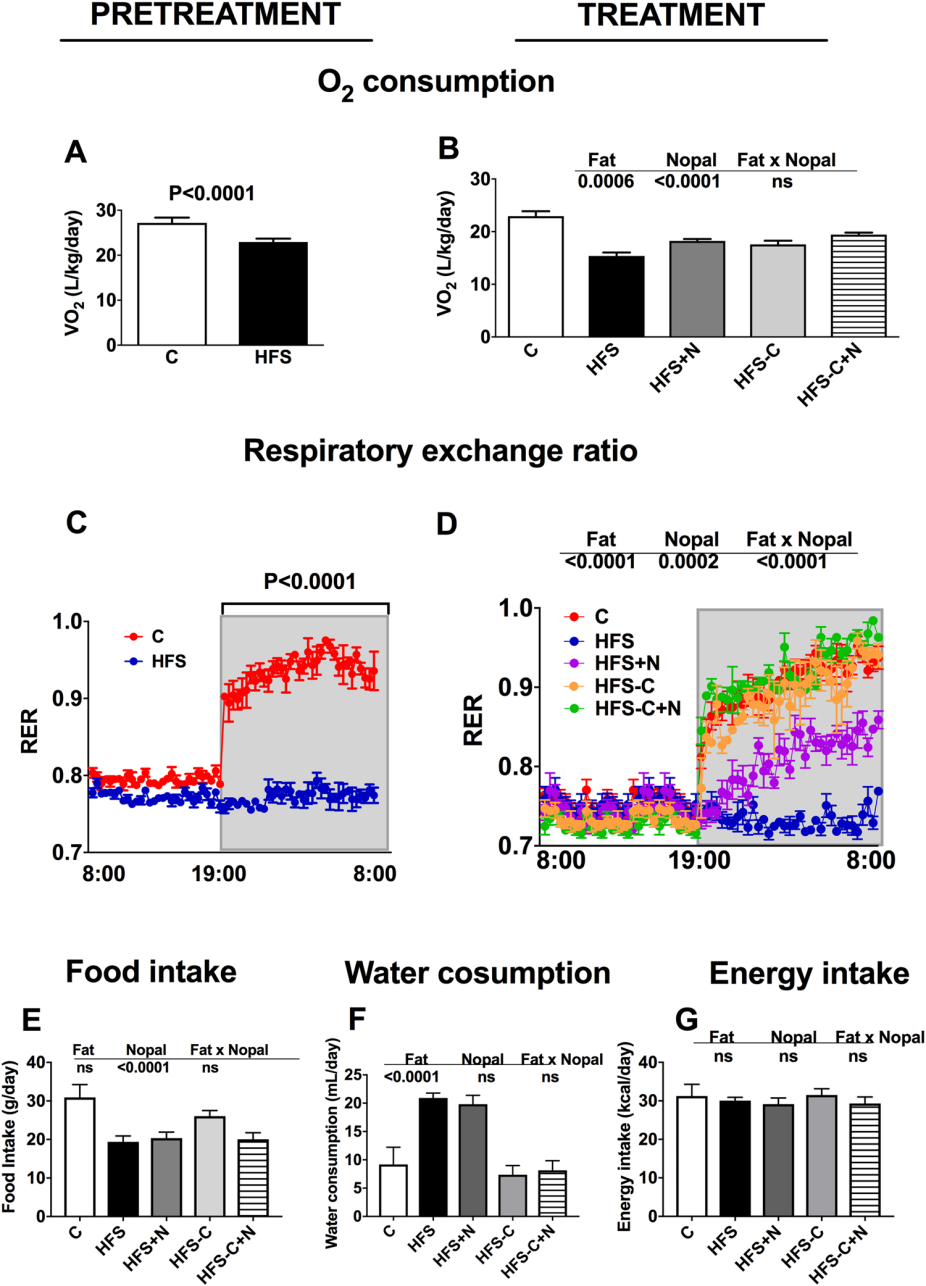
Effect of nopal on Energy expenditure and energy intake. (**A**) O_2_ consumption, (**C**) respiratory exchange ratio of Wistar rats fed a control diet or a high fat diet +5% sucrose in drinking water. water (HFS) during 7 months (pretreatment period). After this period, obese rats were switched to one of the experimental diets for 1 month (treatment period): HFS, HFS + N, HFS-C, HFS-C + N; (**B**), (**D**). Food intake, water consumption and energy intake in all the experimental groups. Values are shown as means ± SEM, n = 5 rats per group. Comparisons among groups were analyzed by two-way ANOVA. Comparisons between 2 groups were analyzed by student t test.

We fed rats with a control diet (C) or high fat-sucrose diet (HFS) for 7 months (pre-treatment period). As expected, rats fed the HFS diet showed significant weight gain (23.6%) and higher concentrations of serum glucose (50.2%), insulin (111.4%) (Fig. 1A,B,D), TG (55.2%), TC (46.3%) and LDL-C (123.5%) compared with the C group (Fig. 2A,C,E). The increase in weight gain in rats fed the HFS diet was accompanied by a significant reduction in energy expenditure (Fig. 3A), in addition to an increase in metabolic inflexibility (Fig. 3C) associated with the use of fatty acids instead of carbohydrates (respiratory exchange ratio) (RER) = 0.778–0.799 as energy substrate^14^. Interestingly, at the end of the one month treatment period, the HFS + N, HFS-C and HFS-C + N groups lost 127.9 g, 148.5 g and 203.9 g of body weight respectively compared with the HFS group and significantly decreased all abnormalities in biochemical parameters (Figs 1A,C,E and 2B,D,F). Likewise, the HFS + N, the HFS-C and HFS-C + N groups showed a significant increase in O_2_ consumption by 13.9%, 14.2% and 22.2% respectively, accompanied by an attenuation in metabolic inflexibility with RERs of 0.87, 0.81 and 0.99 (P < 0.0001) respectively compared with the HFS group (Fig. 3B,D). Although the groups HFS and HFS + N showed lower food intake but higher sweetened water consumption than the rest of the groups, there was no significant difference in energy intake among groups (Fig. 3E–G).

### Nopal consumption modifies the gut microbiota improving the integrity of the intestinal epithelium by reducing metabolic endotoxemia

**Figure 4:**
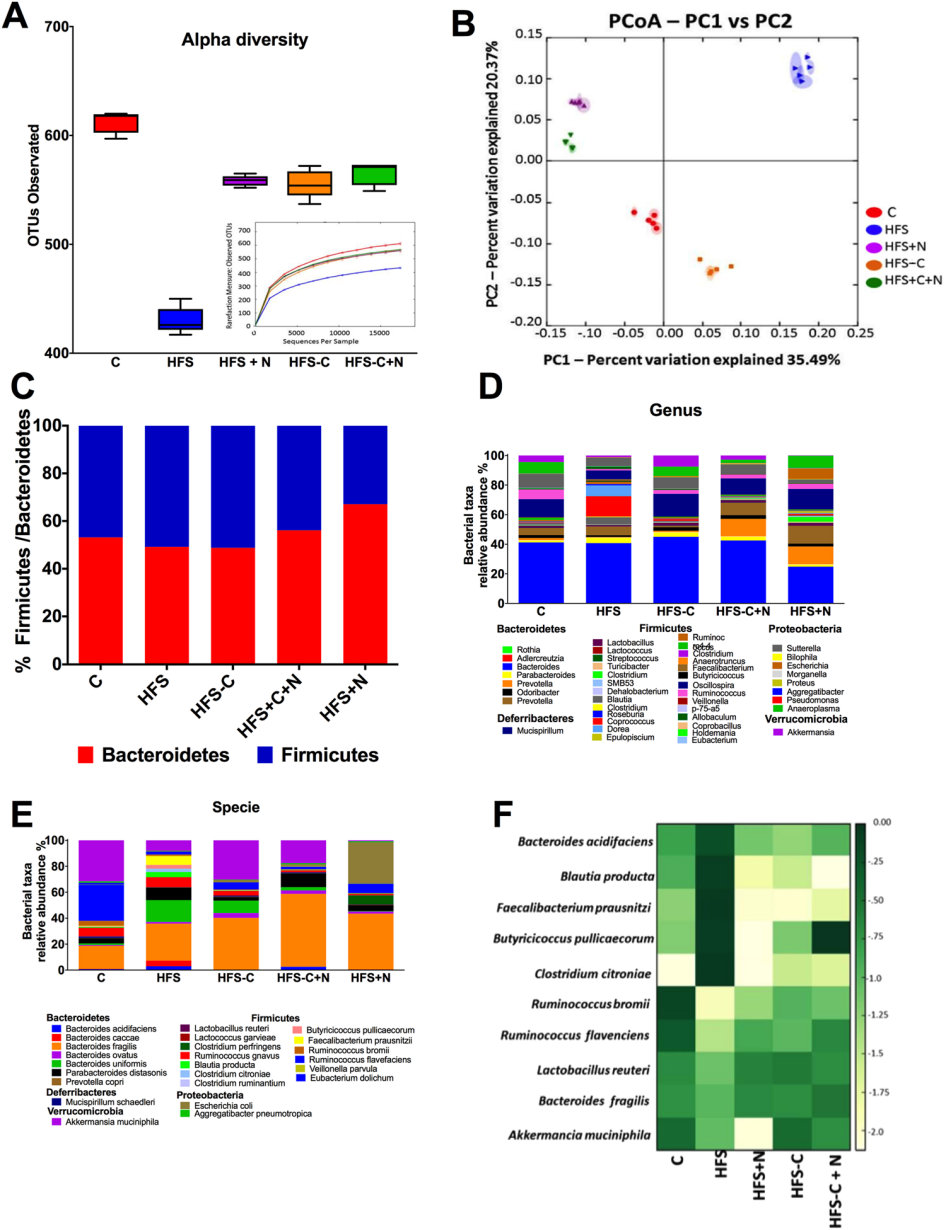
Nopal consumption modifies the intestinal microbiota. (**A**) Alpha diversity and rarefaction curves. (**B**) Principal components analysis of the (**C**), HFS, HFS + N, HFS-C + N and HFS-C groups, n = 5 per group, (**C**) Relative abundance of *Firmicutes* and *Bacteriodetes*, (**D**) relative abundance of gut microbiota at the genus level, (**E**) and at species level and (**F**) heatmap of the ten bacterial species with greatest differences among groups.

**Figure 5:**
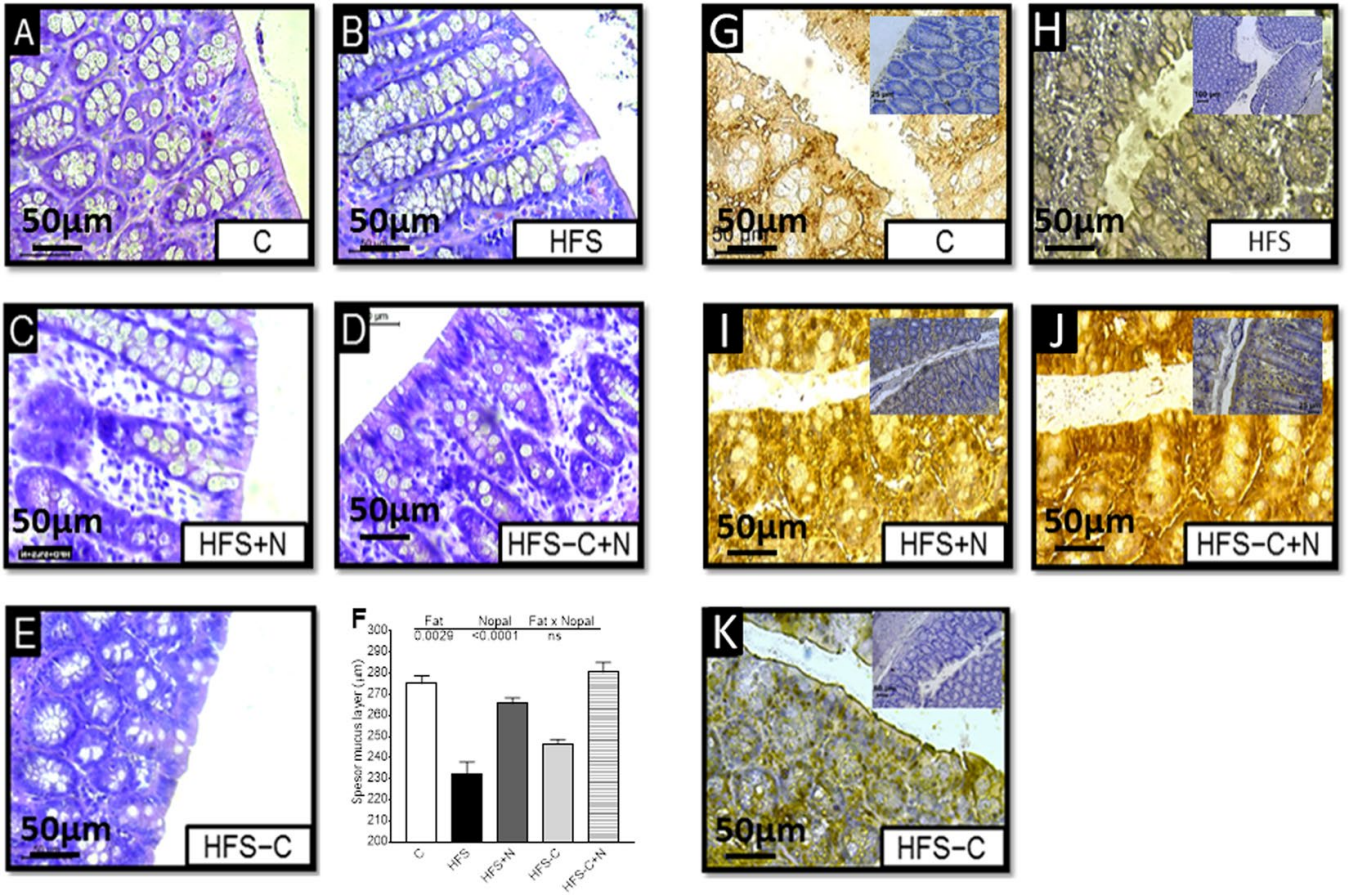
Effect of nopal consumption on histological morphology and expression of occludin-1 in the colon of Wistar rats fed different dietary treatments. (**A**–**E**) Hematoxilin-Eosin staining (HE), (**F**) Mucosal layer quantitative analysis. Values are shown as means ± SEM, n = 5 rats per group and (**G**–**K**). Immunohistochemical analysis of occludin-1 in the colon of rats fed control diet (**C**), high fat diet with 5% sucrose in their drinking water (HFS), obese rats consuming HFS + 5% nopal (HFS + N), and obese rats switched to C diet with (HFS-C + N) or without (HFS-C) 5% nopal at the end of the treatment period 400X magnification.

Since nopal contains soluble and insoluble fibres, as well as several polyphenols, we studied whether the beneficial effects of nopal were associated with changes in the gut microbiota. The relative abundance of the main three phyla *Bacteroidetes*, *Firmicutes and Proteobacteria* represented approximately 93.8% of the sequences at the phylum level. Alpha diversity was estimated by observed OTUs and Chao 1 and Shannon indexes with similar results, indicating that C group showed the highest diversity followed by the HFS + N, HFS-C and HFS-C + N groups, whereas the HFS group had the lowest observed OTUs (P < 0.0001) (Fig. 4A). These results were confirmed using rarefaction curves analysis (inset Fig. 4A) indicating that the dietary treatments increased the specie richness and diversity compared with the HFS group. Clustering the bacterial communities using principal component analysis (PCoA) revealed that the microbiota after nopal consumption (HFS + N, HFS-C + N) was different to that of the HFS, C and HFS-C groups (ANOSIM R = 0.98, P = 0.001) (Fig. 4B). At the phylum level, the analysis revealed that the abundance of *Bacteroidetes* increased with respect to the *Firmicutes* after the consumption of nopal (Fig. 4C). At the genus level, nopal consumption increased *Anaeroplasma*, *Prevotella and Ruminucoccus* by 54.6%, 34.6% and 3.7% respectively and reduced *Faecalibacterium*, *Clostridium* and *Butyricicoccus by* 97.2%, 98.9% *and* 98.8% respectively with respect to the HFS group (Fig. 4D). At the specie level, *Bacteroides fragilis* increased by 14.6% and 27.7% in the HFS + N and HFS-C + N groups respectively compared with the HFS group, whereas the HFS-C group showed an increase in *Akkermansia muciniphila* by 22% compared to the HFS group, (Fig. 4E). A heat map was created based on the species that differed the most among the groups (Fig. 4F). The top five species that increased with the dietary treatments were *Ruminococcus bromii*, *Rumminococcus flavefaciens*, *Lactobacillus reuteri*, *Bacteroides fragilis* and *Akkermansia muciniphila* whereas the top five species that decreased with nopal consumption were *Bacteroides acidifaciens*, *Blautia producta*, *Faecalibacterium prausnitzii*, *Butyricicoccus pullicaecorum* and *Clostridium citroniae*. Interestingly, we observed that rats fed the HFS diet decreased the intestinal mucus layer thickness compared with the control group (Fig. 5A,B,F), this was accompanied by a decrease in the abundance of occludin in the HFS group (Fig. 5G,H). Nopal consumption restored the mucus layer (Fig. 5C,D,F) and increased the abundance of occludin (Fig. 5I,J). The HFS-C group restored the occludin abundance but in a lesser extent (Fig. 5E,K). There is evidence that an improvement in the intestinal permeability reduces the metabolic endotoxemia by decreasing the translocation of the lipopolysaccharide (LPS). The increase in circulating levels of LPS has been associated with the onset and progression of inflammation and metabolic diseases^15^.

### Nopal consumption reduces metabolic endotoxemia, serum GIP and glucose tolerance

**Figure 6:**
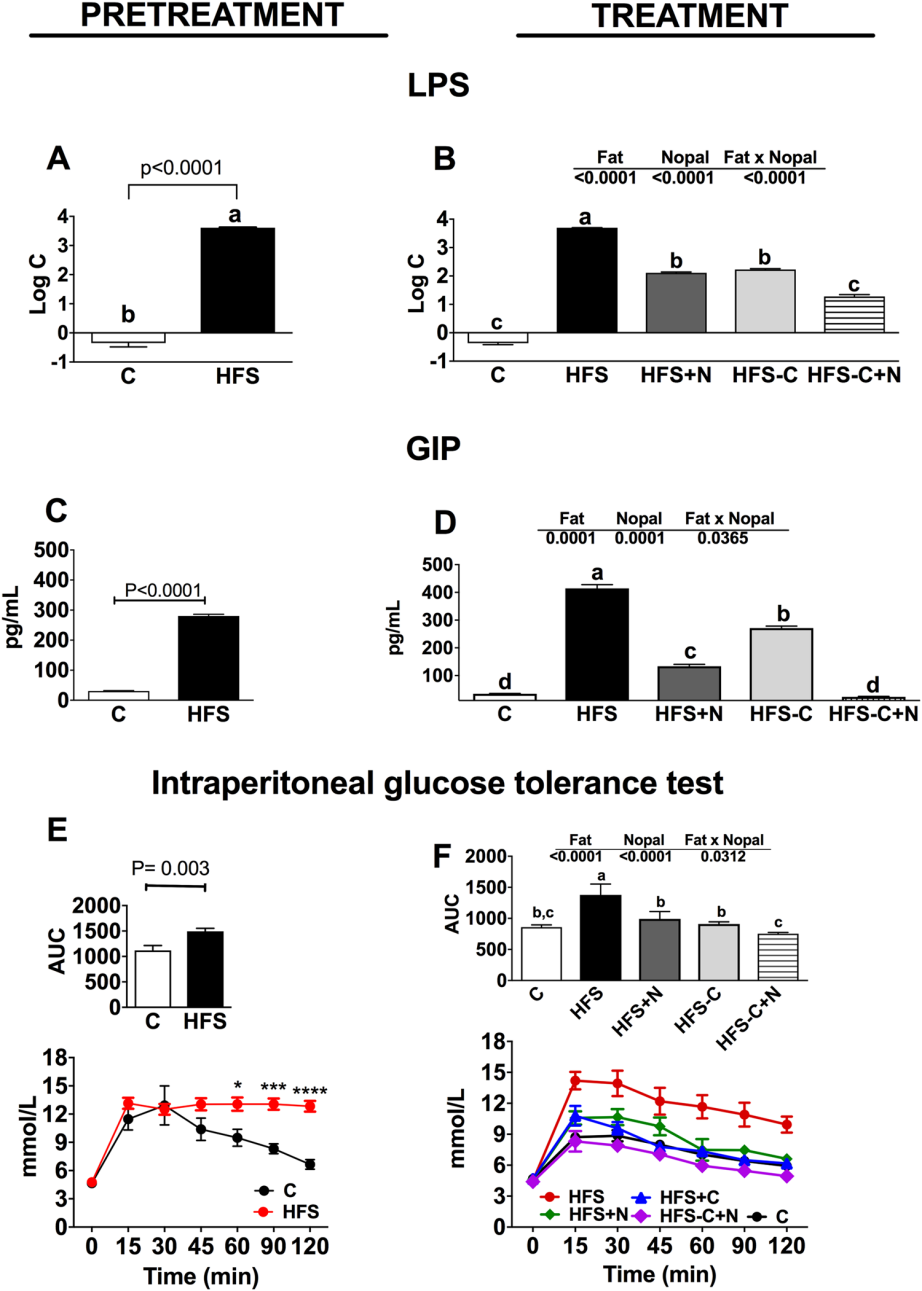
Nopal consumption decreases metabolic endotoxemia, prevents the hypersecretion of glucose insulinotropic peptide (GIP) and improves glucose tolerance. Fasting serum lipopolysaccharide (LPS) after the pretreatment (**A**) and treatments (**B**) periods. Serum GIP after the pretreatment (**C**) and treatments (**D**) periods. ipGTT and area under curve (AUC) after the pretreatment (**E**) and treatments (**F**) periods. Wistar rats were fed one of the following diets: control diet (**C**), high fat diet with 5% sucrose in their drinking water (HFS), HFS + 5% nopal (HFS + N), and obese rats switched to **C** diet with (HFS-C + N) or without (HFS-C) 5% nopal. Data are expressed as mean ± SEM (n = 5/group). Comparisons among groups were analysed by 2-way ANOVA followed by Fisher’s PLSD test. Different letters indicate significant differences among groups (a > b > c > d). P < 0.05. Comparisons between two groups were analysed by student t test.

In order to assess whether the improvement in metabolic parameters were in part associated with metabolic endotoxemia, we measured the circulating levels of LPS. After the pretreatment period for 7 months with the HFS diet, serum levels of LPS increased by 6708-fold compared to the C group (Fig. 6A). By the end of the treatment period, the serum LPS concentration of the HFS group further increased 11132-fold compared with the control group (Fig. 6B), indicative of high-grade metabolic endotoxemia. Interestingly, serum LPS of the HFS + N, HFS-C + N or HFS-C groups showed a dramatic reduction of 97%, 99.5% and 95.6% respectively (Fig. 6B) compared with the HFS group, suggesting that the improvement in the intestinal epithelium by the dietary treatments, reduced circulating LPS levels.

Other possible mechanism by which nopal ameliorates biochemical abnormalities is by modulation of incretins. It has been suggested that modulation of gut peptides could control glucose homeostasis by a mechanism involving gut microbiota^16^. Thus, we measured the effect of nopal on circulating levels of GIP, an incretin released in response to glucose or fat. During the pretreatment period, the HFS group showed 9.2-fold higher serum GIP than those fed C diet (Fig. 6C). Similarly, to LPS, consumption of HFS diet during the treatment period further increased serum GIP by 48% (Fig. 6D). Interestingly, obese rats fed HFS + N and HFS-C + N, or HFS-C showed a significant decrease in GIP by 68%, 94% and 34.6% respectively compared with the HFS group, indicative of a more potent effect of nopal on GIP (Fig. 6D). These results suggest that part of the mechanism by which nopal ameliorates the biochemical abnormalities in obese rats fed HFS involves a reduction of LPS and GIP levels that can be in part associated with changes in gut microbiota^17^. Several studies indicate that a reduction of LPS and GIP can improve glucose tolerance. Thus, we performed an intraperitoneal glucose tolerance test after the pretreatment period. As expected, rats fed the HFS diet showed glucose intolerance and an area under the glucose curve (AUC) that was 32.5% greater than the control group (Fig. 6D). Notably, obese rats fed HFS + N, HFS-C + N or HFS-C during the treatment period of 1 month showed a 26.5% (P < 0.03), 43.8% (P < 0.05) and 31.6% (P < 0.015) reduction, respectively, in the area under the glucose curve compared with the HFS group, indicative that glucose intolerance was reverted after the dietary treatments (Fig. 6E).

### Nopal down-regulated the expression of genes involved in inflammation and oxidative stress in adipose tissue

**Figure 7:**
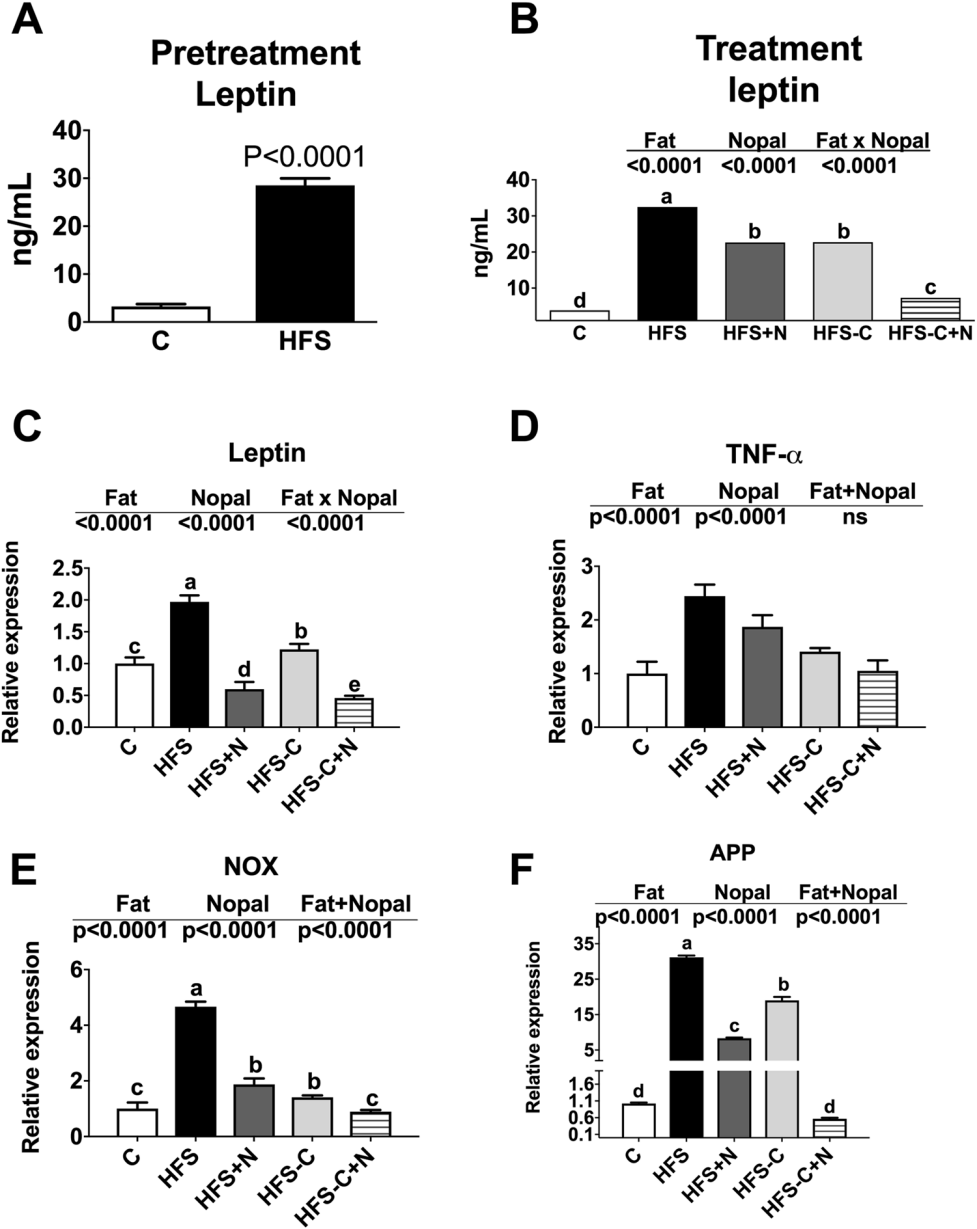
Effect of nopal consumption on serum leptin and expression of genes of inflammation and oxidative stress in adipose tissue. Panel (A) and (B) show the concentration of fasting serum leptin after the pretreatment and treatment periods respectively. Relative mRNA abundance of (**C**) leptin, (**D**) *Tnf-α*, (**E**) NADPH oxidase (*Nox*) and (**F**) amyloid precursor protein (*App*) in adipose tissue of Wistar rats fed one of the following diets: control diet (**C**), high fat diet with 5% sucrose in drinking water (HFS), HFS + 5% nopal (HFS + N), and obese rats switched to C diet with (HFS-C + N) or without (HFS-C) 5% nopal. Data are expressed as mean ± SEM (n = 5/group). Comparisons among groups were analysed by 2-way ANOVA followed by Fisher’s PLSD test. Different letters indicate significant differences among groups (a > b > c > d), P < 0.05. Comparisons between two groups were analysed by student t-test.

It has been demonstrated that some polyphenols can reduce the inflammatory response mediated by LPS^18^. Nopal contains polyphenols that could modulate the expression of genes of inflammation and oxidative stress. We found that obese rats fed HFS + N, HFS-C + N or HFS-C significantly decreased the expression of the genes *Tnf-α*, and NADPH oxidase (*Nox*) involved in inflammation and oxidative stress in white adipose tissue with respect to the rats fed HFS diet, (Fig. 7D,E). Additionally, the pretreatment of rats with HFS diet increased serum leptin concentration by 8.7-fold compared to the C group (Fig. 7A). Interestingly, after 1 month of dietary treatment with HFS + N, HFS-C or HFS-C + N significantly reduced the expression of leptin in adipose tissue by 69.6%, 37.5% and 76.7% with respect to the HFS group (Fig. 7C). Serum leptin concentration during the treatment period decreased by 30.3%, 30% and 77.5% in the groups fed HFS + N, HFS-C and HFS-C + N respectively (Fig. 7B) compared to rats fed HFS diet.

On the other hand, it has been reported that amyloid protein precursor (APP) is related with inflammatory changes in brain and adipose tissue during obesity and is associated with insulin resistance^19^. We observed a 30-fold increase in adipose tissue *App* expression in rats fed the HFS diet for the treatment period compared with the control group, whereas, *App* expression was significantly reduced in the HFS + N and HFS-C + N and HFS-C groups by 73% 98% and 39% respectively, compared with the HFS group (Fig. 7F).

### Consumption of nopal decreases brain oxidative stress, neuroinflammation and improves cognitive function

**Figure 8:**
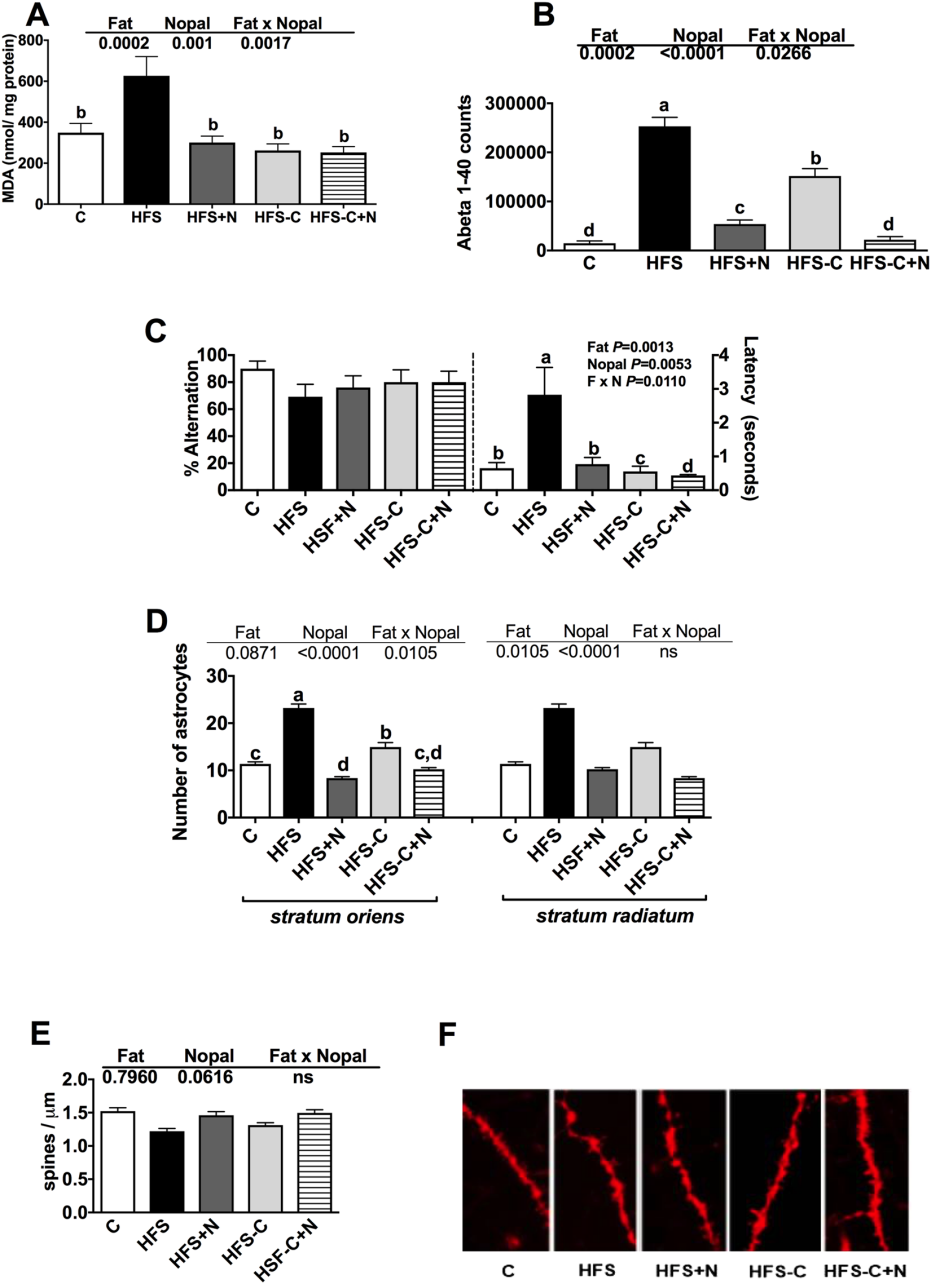
Nopal consumption decreases oxidative stress and improves cognitive function. (**A**) Malondialdehyde (MDA) levels in the prefrontal cortex, (**B**) abeta_1–40_ peptide in the ventral CA1 region, (**C**) spontaneous alternations and latency time, (**D**) glial fibrillary acidic protein (GFAP) in the *stratum oriens* and the *stratum radiatum*, (**E**) spine density in dendrites of the CA1 region, and (**F**) morphology of dendritic spines in the CA1 region. Data are expressed as mean ± SEM (n = 5/group). Comparisons among groups were analysed by 2-way ANOVA followed by Fisher’s PLSD test. Different letters indicate significant differences among groups (a > b > c > d).

It is not known how APP is processed in adipose tissue, however its cleavage products Aβ_1–40_ and Aβ_1–42_ peptides are found in serum^20, 21^. Additionally, these peptides can cross the blood-brain barrier and are present in the brain^22^. Recent evidence demonstrates that these peptides can generate oxidative stress by interacting with iron or cupper ions producing H_2_O_2_^23^. Interestingly, Aβ_1–40_ Amyloid beta peptide particle counts were elevated at the end of the treatment period in rats fed the HFS diet compared with control group (P < 0.0001), (Fig. 8B). Remarkably, the HFS + N and HFS-C + N groups showed a significant decrease in Aβ_1–40_ particles compared with the HFS group (P < 0.0001) (Fig. 8B). Similarly, malondialdehyde (MDA) concentration, a marker of oxidative stress, was significantly increased in the prefrontal cortex of rats fed the HFS diet for the treatment period compared with the control group (Fig. 8A). Dietary treatments returned MDA concentrations to control values (P = 0.0017) (Fig. 8A). While elevation of APP and its cleavage products Aβ_1–40_ are associated with changes in cognitive performance, there were no significant differences in T-maze spontaneous alternation among groups (Fig. 8C). However, latencies to enter to a chosen arm were improved in the HFS-C, HFS-C + N and HFS + N groups compared with the HFS group (P < 0.001) (Fig. 8C), indicating that nopal supplementation had a beneficial effect on cognitive performance of obese rats similar to that of the control group. The group fed the HFS diet showed neuroinflammation with an increase in the number of activated astrocytes in both *stratum oriens* and *stratum radiatum* of the ventral CA1 region of the hippocampus (P < 0.0001) compared with the control group. Nopal supplementation during the treatment period reduced this neuroinflammation compared with the HFS group (P < 0.0001) (Fig. 8D). We quantified spine number in basal dendrites of hippocampal pyramidal neurons of the ventral CA1 region of the hippocampus. The group fed the HFS diet for the treatment period had significantly fewer dendritic spines along basal dendrites compared with the control group (P < 0.0001) and the dietary treatment with nopal significantly restored the number of dendritic spines to control levels (P < 0.05) groups (Fig. 8E and F).

### Nopal decreases inflammation and expression of genes involved in hepatic lipogenesis

**Figure 9:**
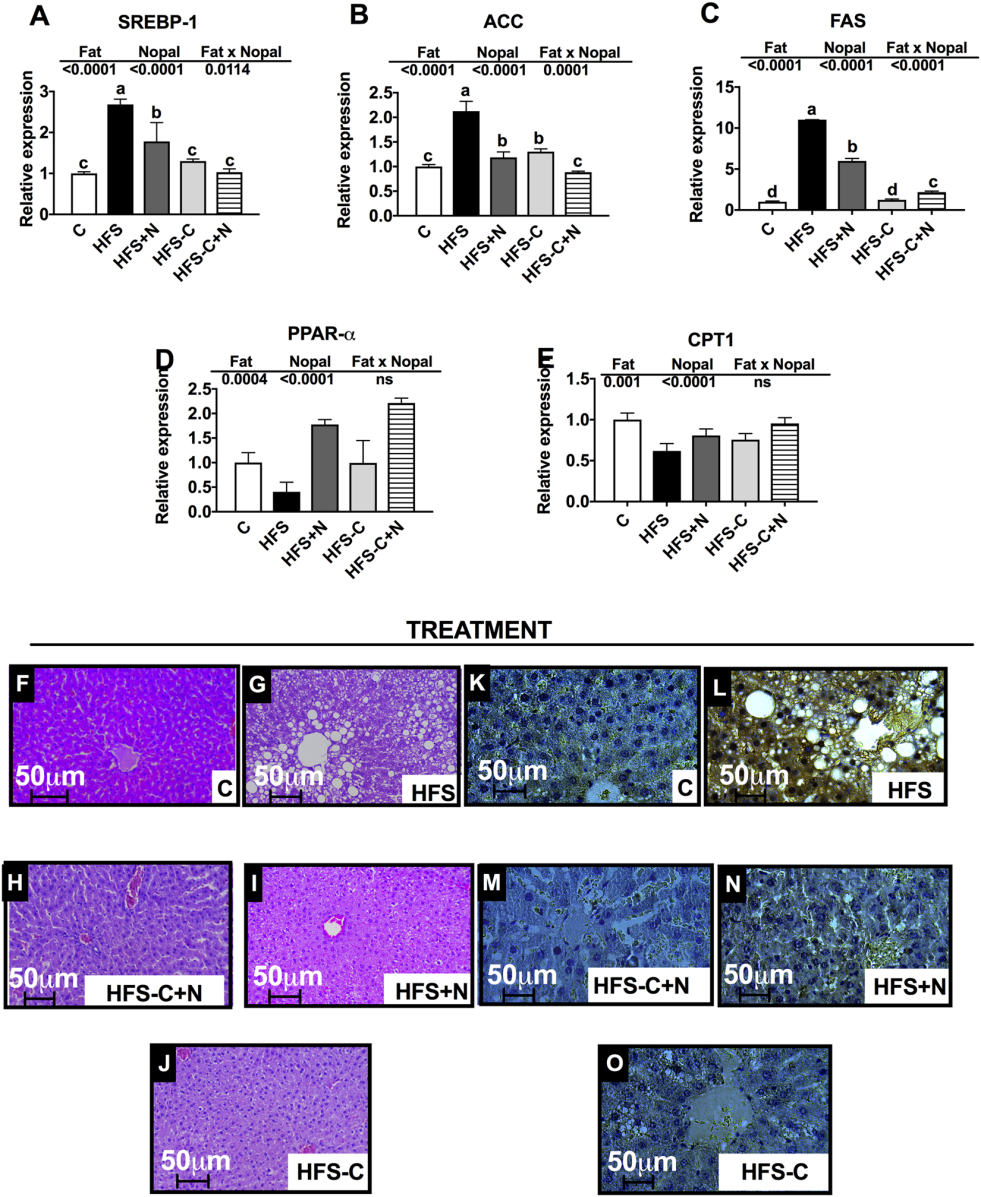
Nopal consumption decreases lipogenesis, hepatic steatosis and inflammation. Relative expression of (**A**) *Srebp-1*, (**B**) Acetyl-CoA carboxylase *Acc*, (**C**) *Fas*, (**D**) *Ppar-α* and (**E**) *Cpt-1* in liver of Wistar rats fed one of the following diets: control diet (**C**), high fat diet with 5% sucrose in drinking water (HFS), HFS + 5% nopal (HFS + N), and obese rats switched to **C** diet with (HFS-C + N) or without (HFS-C) 5% nopal. (**F**–**J**) Hematoxilin-Eosin staining (HE) and (**K–O**) immunohistochemistry of TNF-α in liver of these rats. Data are expressed as mean ± SEM (n = 5/group). Comparisons among groups were analysed by 2-way ANOVA followed by Fisher’s PLSD test. Different letters indicate significant differences among groups (a > b > c > d). 400X magnification.

Finally, since rats fed a HFS showed glucose intolerance and high serum insulin concentration, a condition that stimulates lipogenesis, we studied the expression of genes involved in lipid metabolism in liver. The results showed that the amelioration in the biochemical abnormalities of serum glucose and lipids in the HFS + N, HFS-C + N and HFS-C groups was accompanied by a reduction in the expression of genes involved in lipogenesis. The expression of the sterol regulatory element-binding protein-1 (*Srebp-1*), fatty acid synthase (*Fas*) and acetyl CoA carboxylase (ACC) was reduced by 33%, 45% and 46%, respectively, in the HFS + N group, by 62%, 58% and 80%, respectively, in the HFS-C + N group and by 51%, 89% and 37%respectively, in the HFS-C group (Fig. 9A,B,C). In addition, nopal consumption significantly increased the mRNA abundance of the transcriptional factor Peroxisome proliferator activated receptor *(Ppar)-α* involved in fatty acid oxidation (Fig. 9D) including its target gene, carnitine palmitoyltransferase- 1 (*Cpt-1*) (Fig. 9E). As a consequence, consumption of the HFS diet for the treatment period resulted in the accumulation of lipid droplets in the liver and inflammation with respect to the control group (Fig. 9F,G,K,L). However, the dietary treatments significantly decreased hepatic steatosis (Fig. 9H,I,J) and inflammation mediated by tumor necrosis factor-α (*Tnf-α*) (Fig. 9M,N,O) with respect to the HFS group.

## Discussion

Obesity is a world-wide public health problem that is accompanied of metabolic alterations that includes, hyperglycemia and insulin resistance among others. Several dietary strategies have been developed to control the biochemical abnormalities using plant-derived foods. There is evidence that the consumption of nopal, a vegetable widely consumed in Mexico and south part United States reduces the postprandial blood glucose peaks, serum insulin and GIP in healthy and subjects with type 2 diabetes^12^. However, it is less known about the possible mechanism of action of nopal during obesity. In the present study, obese rats fed a HFS diet with nopal significantly decreased most of the biochemical abnormalities associated with obesity to levels similar to those fed the control diet. Interestingly the addition of nopal in the diet further decreased specific biochemical parameters such serum triglycerides, total cholesterol and GIP. These results can be attributed in part to the low glycemic index of nopal^12^, as well as the presence of polyphenols, soluble and insoluble fibre that modify gut microbiota. As a consequence, there is an antihyperglycemic effect of nopal in the obese rats that can be due in part to a reduction in serum LPS and GIP. In addition, we observed an increase in the alpha-diversity of gut microbiota after the consumption of nopal. At the genus level, *Prevotella* was significantly decreased in the HFS group, however, obese rats fed HFS + N or HFS-C + N for 1 month increased the abundance of the genus by 11.6- and 9.3-fold, respectively. The presence of *Prevotella* is associated with plant-rich diets and with an improvement of glucose tolerance^24^. We observed that obese animals fed with nopal significantly reduced glucose intolerance (p > 0.0001) and insulin resistance similar to the control group. *P copri* possesses a number of enzymes and gene clusters essential for fermentation and utilization of complex polysaccharides. It is likely that changes at the smaller community level have a significant impact on disease progression, therefore, we assessed whether a HFS diet affected the abundance of other types of bacteria. Interestingly, the HFS + N and HFS-C + N groups showed increases in *B*. *fragilis* of 43.1% and 56.2%, respectively, compared with the HFS or HFS-C group. It has been demonstrated that *B*. *fragilis* regulates intestinal permeability and metabolic homeostasis, preventing leakage of harmful molecules from the gastrointestinal lumen to the circulation^25^. An increase in *B*. *fragilis* has been associated with an increase in the expression of occludin, which are important in maintaining the tight junctions in the epithelial cells of intestine diminishing the leakage of LPS into the circulation^25^. Normally, LPS in the gut does not penetrate the healthy intestinal epithelium, however, we found that after long term consumption of a high saturated fat diet, the low abundance of occludin in tight junctions assembly and maintenance can allow paracellular flux of LPS called metabolic endotoxemia^26^. Here, we found that long term (7 months) consumption of a combination of high fat-sucrose diet by rats produced extremely high serum LPS concentrations (4985 ng/mL) compared with those of control rats (0.45 ng/mL). We found that obese rats fed HFS + N remarkably reduced by 97% circulating levels of LPS similar to the group HFS-C and even more (99.6%) in the group fed HFS-C + N.

It is known that excessive fat or glucose intake induces hypersecretion of GIP^27^, leading to hyperinsulinemia followed by fat accumulation and obesity. Interestingly, inclusion of the nopal in HFS diet for 1 month resulted in a significant decrease in GIP and insulin concentrations, indicating that nopal can prevent GIP hypersecretion and hyperinsulinemia. Mice lacking the GIP receptor that are fed a high fat diet are shown to be protected from both obesity and insulin resistance^27^. The inclusion of nopal in the diet in the present study significantly decreased glucose intolerance as a result of the decrease in insulin resistance. We suggest this was partly caused by the presence of complex carbohydrates in nopal, which take longer to digest and have less impact on blood glucose, causing it to rise more slowly in addition to changes in the gut microbiota. Interestingly, we found that serum GIP concentrations above 400 pg/µl showed an exponential increase in LPS. Moreover, high LPS concentrations are a trigger for obesity and diabetes and induce leptin synthesis^28^, as we observed in our rats fed the HFS diet. Increases in LPS and leptin induce expression of the pro-inflammatory cytokine TNF-α in liver. The consequent insulin resistance favours hyperinsulinemia and excessive hepatic and adipose tissue lipid storage as observed in our HFS-fed rats. It has been suggested that the fat content of food is an important regulator of plasma LPS concentration^29^, which accelerates the progression of nonalcoholic hepatic steatosis^30^. In the present study, in rats fed the HFS diet we observed a marked accumulation of lipid droplets in the liver indicating hepatic steatosis and a greater expression of SREBP-1 and FAS in the liver, compared with the control group. Interestingly, inclusion of nopal in the diet significantly decreased LPS, leptin and the abnormalities produced by the long-term consumption of a HFS diet.

**Figure 10:**
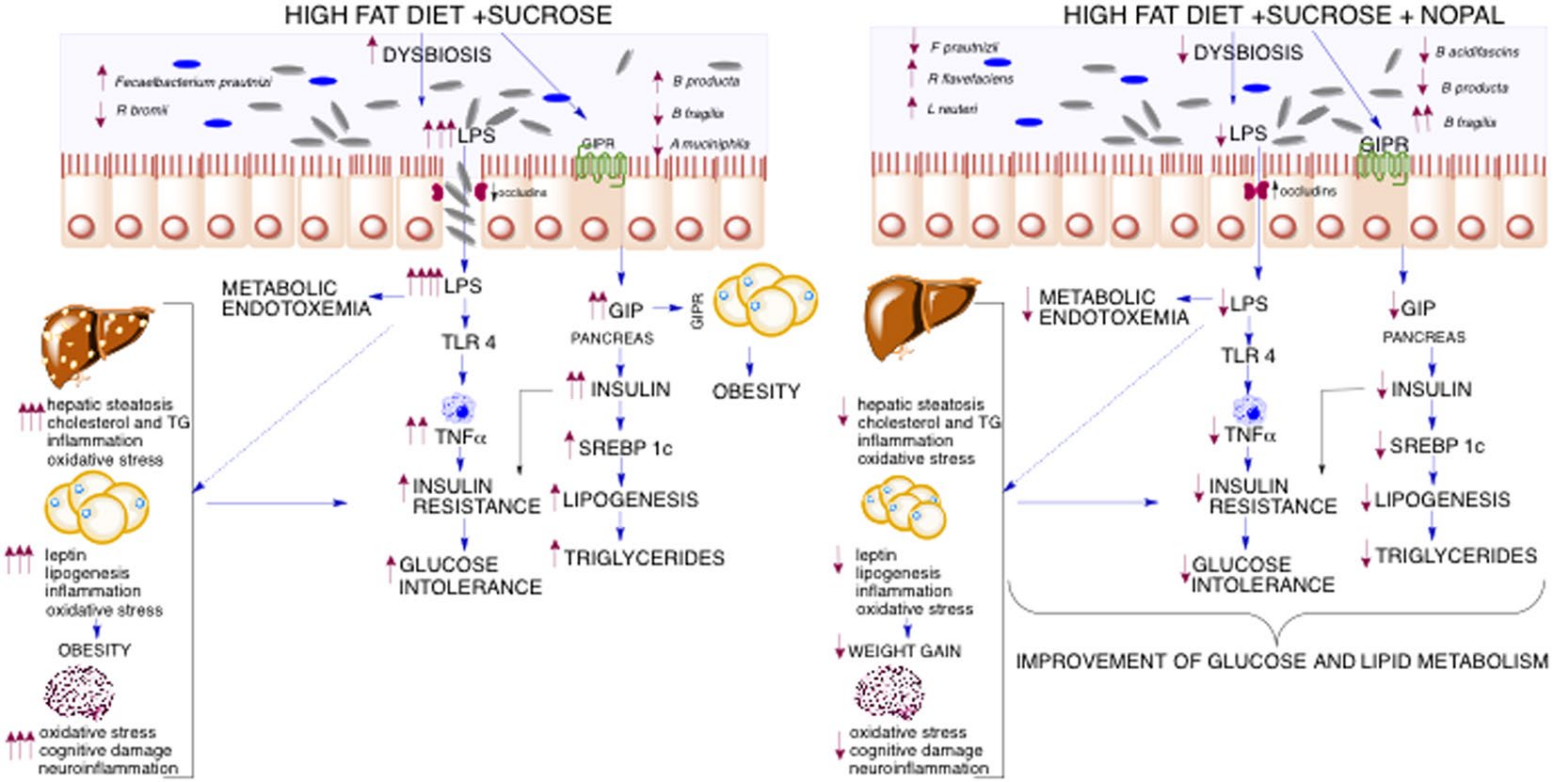
Consumption of nopal, a vegetable rich in soluble and insoluble fibres, polyphenols, vitamin C and with a low glycemic index, reduces gut dysbiosis and increases the abundance of occludin-1 improving intestinal permeability associated with an increase in *B fragilis* which in turn reduces paracellular transport of lipopolysaccharide (LPS) by the intestinal epithelium reducing metabolic endotoxemia. Nopal consumption also decreases the hypersecretion of glucose insulinotropic peptide (GIP). The metabolic consequences are a reduction in insulin resistance, lipogenesis, hepatic steatosis and cognitive damage. This figure was created using the software ChemBio Draw v. 13.0.2.3020 (www.cambridgesoft.com).

Finally, our data demonstrated a significant negative correlation between the abundance of *B*. *fragilis* and circulating levels of serum GIP, insulin, LPS, the number of positive astrocytes for Glial Fibrillary Acidic Protein (GFAP) in the *stratum oriens* and *stratum radiatum*, and the concentration of MDA in the prefrontal cortex (Supplementary Fig. S1A–F). As a consequence, there was a significant positive correlation between the percentage alternation and the abundance of *B*. *fragilis* (Supplementary Fig. S1G), leading in a significant decrease in latency (Supplementary Fig. S1H). From these correlations, we show that the consumption of nopal greatly increases the abundance of *B*. *fragilis*, improving glucose and lipid metabolism, decreasing metabolic endotoxemia and improving cognitive function, despite the consumption of a HFS diet (Fig. 10). The anti-inflammatory effect of nopal is particularly interesting, since several studies suggest that the gut microbiota is involved in the development of low-grade inflammation associated with obesity^15^. We observed that both oxidative stress and neuroinflammation in the brain were reversed by dietary nopal supplementation. It has been postulated that consumption of a HFS diet affects the permeability of the blood-brain barrier, allowing free passage of proinflammatory cytokines and other molecules (i.e. APP and Aβ_1–40_ peptides) causing impairment in hippocampal-dependent memory tasks^5^. Recent studies highlight the relation between gut microbiota and brain, in particular, obesity-induced dysbiosis has been associated with poor cognitive flexibility^9^, highlighting the role of microbiota on central nervous system function.

In conclusion, the results of the present work indicate that modifications in the diet can reduce several abnormalities in biochemical parameters produced by obesity, interestingly the addition of nopal can ameliorate specific biochemical paremeters of obesity such as total cholesterol, GIP, APP, leptin and Aβ_1–40_ peptides modifying the gut microbiota despite the consumption of a HFS diet, generating metabolic benefits similar to those observed when obese rats are switched to consume a C diet. Finally, the inclusion of nopal in a healthy diet can further reduce in a greater extent most of the abnormalities in biochemical parameters. Further clinical studies are needed to establish whether this beneficial health effects can be observed in humans.

## Methods

### Diets and animals

Diets were administered in dry form. The control diet (C) was prepared according to the recommendations for AIN-93^31^. The high fat-sucrose diet (HFS) consisted of a high fat diet (45% kcals from fat) and 5% sucrose added to the drinking water. The nopal-supplemented diets were formulated to provide 5% of dietary fibre from nopal in place of cellulose. The carbohydrate and protein content of the experimental diets were adjusted for the content of these nutrients provided by the dehydrated nopal (Supplementary Table 1). To study the effect of nopal in a diet induced obesity model, male Wistar rats from approximately 6 weeks age from the animal care facility with a weight of approximately 200 g were housed in individual cages in a room with controlled temperature (22 °C) and humidity and a 12-h light/dark cycle. Rats were divided in two groups for the pretreatment period (7 months): a control group (n = 5) fed the C diet, and a high fat-sucrose group (n = 20) fed the HFS diet. Immediately following the pretreatment period, rats from the high fat-sucrose group were randomly assigned to one of four groups for the treatment period (1 month), fed the following diets (n = 5 per group): (1) HFS (HFS group), (2) C (HFS-C group), (3) HFS + 5% dehydrated nopal (HFS + N group), (4) C + 5% dehydrated nopal (HFS-C + N group). The control group from the pretreatment period continued consuming the C diet for the treatment period.

### Dehydrated nopal

Nopal cladodes were obtained from Milpa Alta (Mexico City), diced and dehydrated at 55 °C for 24–36 h to prevent loss of antioxidant activity. The carbohydrate and protein content of the dehydrated nopal replaced maltodextrin and casein in the C diet.

### Food intake, glucose tolerance, and behavioural measurements

Food intake and body weight gain were measured every other day. Glucose tolerance and energy expenditure were measured at the end of the pretreatment and treatment periods. The glucose tolerance test is described in the online Supplementary methods. Behavioural measurements (T-maze spontaneous alternation) were taken 1 week before the end of the treatment period. To assess gut microbiota, fecal samples were collected from three animals from each group at the end of the treatment period and stored at −80 °C. Rats were killed by decapitation after being anesthetized with sevoflurane. Liver, adipose tissue and intestine were rapidly removed. A sample of each was snap-frozen and stored at −70 °C until analysis, and another fixed with ice cold 4% (w/v) paraformaldehyde in a phosphate buffer. Brain was also rapidly removed and treated as described below. Serum was obtained by centrifugation of blood at 1500 × g for 10 min and stored at −70 °C until further analysis. All procedures were carried out in accordance with the international guidelines and regulations for the use of experimental animals, and the protocol was approved by the Animal Committee of the National Institute of Medical Sciences and Nutrition, Mexico City (No. 277).

### Energy expenditure by indirect calorimetry

To measure the type of substrate used at the end of the pretreatment and treatment periods, energy expenditure was measured by indirect calorimetry as described in the online Supplementary methods.

### Metabolic variables

Serum insulin was measured using an insulin radioimmunoassay kit (Millipore, Billerica, MA). Serum parameters including glucose, triglyceride (TG), and total (TC) and LDL cholesterol (LDL-C) were measured using an enzymatic-photometric assay with a Cobas c111 analyzer (Roche Applied Science). Serum leptin was measured using a rat leptin enzyme-linked immunosorbent assay kit (Merck, Millipore, USA). Plasma GIP was measured using a RayBio enzyme immunoassay kit (RayBiotech), and serum LPS was measured using a competitive inhibition enzyme immunoassay (Cloud-Clone Corp, Houston, TX). Formation of malondialdehyde (MDA) in the brain was measured as previously reported^32^.

### Quantitative real-time PCR

Extraction of total RNA from tissues and synthesis of cDNA were carried out as previously described^33^. mRNA abundance was measured by real-time quantitative PCR using the SYBR Premix assay (Roche) as described in the online Supplementary methods.

### Histological analysis

Liver, intestine and adipose tissue sections 4 µm thick were stained with hematoxylin and eosin. A morphological analysis was performed using a Leica microscope (Leica DM750 Wetzlar, Germany).

### Immunohistochemical analysis

Paraformaldehyde-fixed subsamples of liver and colon were dehydrated, embedded in paraffin, and processed as described in the online Supplementary methods.

### Microbiota analysis

#### DNA isolation and sequencing

Fresh fecal samples were collected immediately, frozen and stored at −70 °C until use. Bacterial DNA content was extracted using the QIAamp DNA Mini Kit (Qiagen, Valencia, CA, USA) according to the manufacturer’s instructions.

MiSeq platform was used for the sequencing of the samples and then genomic libraries of the regions V3 and V4 of the 16S gene were generated, using primers for those regions that also contained an overhang adapter specified by Illumina (F: 5′-TCGTCGGCAGCGTCAGATGTGTATAA GAGACAGCCTACGGGNGGCWGCAG-3′ and R: 5′-GTCTCGTGGGCTCGGAGA TGTGTATAAGAGACAGGACTACH VGGGTATCTAATCC-3′) The amplicons of the V3 and V4 regions were generated by PCR reactions containing genomic DNA (5 ng/μL in 10 mM Tris pH 8.5), high Fidelity DNA polymerase 2× KAPA HiFi HotStart ReadyMix and primers (1 μM). This mixture was placed into the thermal cycler and run through the following program: 3 min at 95 °C, followed by 25 amplification cycles consisting of denaturation (30 s at 95 °C), alignment (30 s at 55 °C) and elongation (30 s at 72 °C). The final elongation consisted of 5 min at 72 °C. The amplicons were purified using AMPure XP beads and their size was verified on capillary electrophoresis in the Agilent 2100 Bioanalyzer (Agilent Technologies, Santa Clara, California, USA), with an approximate size of 550 bp. Once passed the quality control, the samples were indexed using the Illumina Nextera XT Index Kit (v.2, Set A). For this process 5 μL of the first PCR product, High Fidelity DNA polymerase 2× KAPA HiFi HotStart ReadyMix and primers (Index) were mixed and returned to the thermocycler, using the following program: 3 min at 95 °C, followed by 8 amplification cycles which consisted of denaturation (30 s at 95 °C), alignment (30 s at 55 °C) and extension (30 s at 72 °C). The final extension consisted of 5 min at 72 °C. This product was purified and the integrity was analyzed. The amplicons had an approximate size of 610 bp. The concentration of double-stranded DNA was determined by fluorometry (Qubit fluorometer 3.0, high sensitive kit). The final library was mixed equimolarly and sequenced on the Illumina MiSeq platform (MiSeq Reagent Kit V.3, 600 cycles) following the supplier’s instructions.

#### Sequence Analysis

For taxonomic composition analysis, Custom C# and python scripts, as well as python scripts in the Quantitative Insights Into Microbial Ecology (QIIME) software pipeline 1.9 were used to process the sequencing files. The sequence outputs were filtered for low-quality sequences (defined as any sequences that are <200 bp or >620 bp, sequences with any nucleotide mismatches to either the barcode or the primer, sequences with an average quality score of <25, and sequences with ambiguous bases >0). Sequences were chimera checked with Gold.fa, and chimeric sequences were filtered out. Analysis started by clustering sequences within a percent sequence similarity into Operational taxonomic units (OTUs). 91% of the sequences passes filtering, resulting in 83,906 sequences/sample with a 97% similarity threshold. Operational taxonomic units (OTUs) picking was performed using tool set Qiime-tools, using Usearch method. OTUs were picked against the GreenGenes 13.9. 97% OTUs reference database. After the resulting OTU results files were merged into one overall table, taxonomy was assigned based upon the gg v13.9 reference taxonomy. Thus, 99.76%, 99.69%, 99.64%, 86.8%, 50.48% and 10.86% of the reads were assigned to the phylum, class, order, family, genus and specie level, respectively. Species richness (Observed, Chao1) and alpha diversity measurements (Shannon) were calculated, and we estimated the within-sample diversity at a rarefaction depth of >17351 reads per sample. Weighted and unweighted UniFrac distances were used to perform the principal coordinate analysis (PCoA).

Microbial sequence data were pooled for OTUs comparison and taxonomic abundance analysis but separated by batch in principle coordinates analysis (PCoA) to have clear PCoA figures. For even sampling, a depth of 17,351 sequences/sample was used. PCoAs were produced using Emperor. Community diversity was determined by the number of OTUs and beta diversity, measured by UniFrac unweighted and weighted distance matrices in QIIME. ANOSIM, a permutational multivariate analysis of variance was used to determine statistically significant clustering of groups based upon microbiota structure distances.

### Cognitive evaluation, glial fibrillar acidic protein (GFAP) and amyloid Abeta_1–40_ peptide immunohistochemistry and morphology of dendrites in the hippocampus

These assays were conducted as described in the online Supplementary methods.

### Statistical analyses

Data are expressed as mean ± standard error of the mean (SEM). Data distribution was assessed by using the Shapiro-Wilk normality test. Statistical analysis of the pretreatment period was assessed by student t-test. Comparisons among groups after the treatment period were analyzed by two-way ANOVA followed by Fisher’s least significant difference post-hoc test. Comparisons between 2 groups were analyzed by t-student test by using Prism statistical software (GraphPad Prism for Mac OSX v.7. Results were considered statistically significant at P < 0.05).

## Electronic supplementary material

supplementary information
