# Supplementary material for: Nopal (Opuntia ficus indica) protects from metabolic endotoxemia by modifying gut microbiota in obese rats fed high fat/sucrose diet
Source: Sci Rep. 2017 Jul 5;7:4716. doi: 10.1038/s41598-017-05096-4 (PMC5498631; doi:10.1038/s41598-017-05096-4)
Supplement: Supplementary file 1 — supplementary information [file 41598_2017_5096_MOESM1_ESM.pdf]

Nopal (*Opuntia ficus indica*) protects from metabolic endotoxemia by modifying gut microbiota in obese rats fed high fat/sucrose diet

Mónica Sánchez-Tapia, Miriam Aguilar-López, Claudia Pérez-Cruz, Edgar Pichardo-Ontiveros, Mei Wang, Sharon M Donovan, Armando R Tovar, Nimbe Torres

SUPPLEMENTARY METHODS

Glucose tolerance test. The glucose tolerance test was performed in all rats by an intraperitoneal administration of a glucose load (2 g/kg) after 6 h of fasting. Blood glucose was determined using a Freestyle Optimum blood glucose monitoring system (Abbot Laboratories, Abbot Park, IL), with blood samples collected from the tail vein at 0, 15, 30, 45, 60, 90, and 120 min after the glucose administration. The area under the curve (AUC) was calculated using the trapezoid rule.

Energy expenditure. Energy expenditure was measured using an indirect respiration calorimetry system (CLAMS, Columbus Instruments, Columbus, OH). Air is drawn through calorimetry chambers and flow rate is controlled and measured with a mass flow controller. Oxygen consumption and carbon dioxide production are measured and used to calculate energy expenditure and respiratory exchange ratio (RER: VCO_2/VO_2). The energy expenditure was monitored in individually housed animals during 48 h in plexiglass cages with open flow system connected to an Oxymax-CLAMS Lab Animal Monitoring System. Animals were acclimatized for 24 h, fasted for 6 h in the light period

and fed during the dark period. Throughout the test, volume of O_2 consumption (VO_2, mL/kg/h) and CO_2 production (VCO_2, mL/kg/h) were measured sequentially during 90 s intervals. The respiratory exchange ratio (RER) was calculated as the average ratio of CO_2 produced to O_2 inhaled (VCO_2/VO_2).

Quantitative real-time PCR.

Total RNA from liver and adipose tissue were extracted according to the method of Chomczynski and Sacchi, followed by determination of integrity, concentration and purity. cDNA synthesis was carried out using an M-MLV reverse transcriptase enzyme and oligo (dT) 12–18 primer (Invitrogen, Carlsbad, CA, USA). The mRNA levels were measured by real-time quantitative PCR using and SYBR Green I® premix assay (Roche Diagnostics, Indianapolis, USA). The PCR scheme used was 50 °C for 2 min, 95 °C for 10 min, and 40 cycles of 95 °C for 15 s followed by 60 °C for 1 min. SYBR Green I® (Sigma-Aldrich, St. Louis, MO, USA) was used according to the manufacturer’s instructions, using cyclophilin and HPRT as the univariate controls. The relative amounts of mRNA were calculated using the $\Delta\Delta Ct$ method with an efficiency adjustment according to the Pfaffl equation.

SYBR Green probes and oligonucleotide primers for peroxisome proliferator activated receptor alpha (PPAR-α), carnitine palmitoyltransferase-1 (CPT-1), sterol regulatory element-binding protein-1c (SREBP 1-c), acetyl-CoA carboxylase1 (ACC 1), fatty acid synthase (FAS), NADPH oxidase (NOX) and tumor necrosis factor alpha (TNF-α). PCR assays for each target gene were analysed in triplicate in a 96-well assay plate with a Light Cycler 480 II (Roche). The relative amount of mRNA was calculated by using the

$\Delta\Delta$Ct method with an efficiency adjustment according to the Pfaffl equation. Cyclophilin and HPRT were used as the invariant control.

Data are presented as the relative transcript levels compared with the cyclophilin and HPRT housekeeping genes, according to the equation for relative expression [1]

Cognitive evaluation.

Five days before finalizing all dietary interventions, animals were tested in the T-maze (spontaneous alternation) [2]. Latency to enter to a chosen arm (seconds) and percentage of alternations between arms (% alternation) were obtained in a total of five sessions during two days per animal[2].

Glial fibrillar acidic protein (GFAP) and Amyloid Abeta$_{1\text{-}40}$ peptide immunohistochemistry

Brain was rapidly removed from skull, kept in 4% paraformaldehyde (PFA) during 5 h, cryoprotected in sucrose (30% p/v), and cut in 40 µm thick slices. Brain slices were incubated with antibodies against GFAP (1:1000, Cell Signalling Technology, Inc. MA, USA) and Abeta$_{1\text{-}40}$ (1:250, Invitrogen, USA) during 24 h at 4ºC and exposed to anti-mouse, or anti-rabbit IgG biotinylated secondary antibody, respectively (1:200, Vector Laboratories, USA) for 120 min at 37°C. All slides were develop using the DAB MapTM Kit (Vector Laboratories, USA) as described by manufacturer and mounted with Vectarshield (Vector Laboratories, USA). Samples with omission of primary antibody were included as negative controls. Light field microscopy (Nikon Eclipse 80i) was used to obtain images (GFAP: 20x objective; Abeta$_{1\text{-}40}$: 100x/oil objective). For GFAP analysis, at least six images of *stratum oriens*, and six images of *stratum radiatum* of

CA1 region were taken per brain slice (3 slices per animal). For Abeta$_{1-40}$ evaluation, immune-reactivity was determined in *stratum pyramidale* of ventral CA1 region, analysing at least three brain slices per animal. Immunopositive signal was counted by use of Image J analysis system (NIH public domain).

Morphology of dendrites in the hippocampus.

Light-fixed brain slices (4% PFA for 5 h) were cut at 150 μm thickness and shot with tungsten particles (1.2 μm) (Bio-Rad, USA) covered with DiL (1,1'-Dioctadecyl-3,3,3',3' Tetramethylindo-carbocyanine Perchlorate, Molecular Probes Life Technologies, USA) by use of an Helius Gene Gun (Bio-Rad, USA). Slices were washed in PBS overnight at 4°C and mounted with glycerol. At least six images per animal were taken from basal dendrites of neurons placed in dorsal CA1 hippocampal region with use of a confocal laser microscope (TCS-SP-8, Leica) under a 100x objective. Same parameters were used for all images (zoom factor=5; average speed 600 Hz; frame average=1; line average=1; frame accumulation =1; line accumulation =1; z stack = 0.1 μm). Z-stack of images were merged and spine quantification in dendritic segments longer than 20 μm was done by use of Image J analysis system (NIH public domain)

Immunohistochemical analysis.

Samples of liver and colon were dissected and immediately fixed with ice-cold 4% (w/v) paraformaldehyde dissolved in phosphate buffer and subsequently dehydrated and embedded in paraffin. Two 4-μm sections per block were stained with hematoxylin and eosin. Deparaffinized tissue sections were blocked with 10% peroxidase/methanol for 10 min, washed with 0.05% PBS-Tween and blocked for 30 min in background sniper solution (Biocare Medical, Concord, CA). For TNF-α immunohistochemical staining, sections were incubated with anti-TNF-α antibody (1:200, EMD Millipore, Temecula, CA) at 4°C overnight. Slides were then incubated during 30 min with mouse Immunodetector Biotin Link (Bio SB, Goleta, CA), incubated for 30 min with mouse immunodetector HRP label (Bio SB, Goleta, CA) and developed during 1 min with diaminobenzidine (DAKO, Glostrup, Denmark) with 0.05% Tween- PBS and with washes in between. Washed slides were counterstained with hematoxylin. All samples were observed under a microscope (Leica DM750 Wetzlar, Germany), photographed with a digital camera (Leica DMC2900), and processed with the imaging software Leica LAS Core V4.5.

References

1 Pfaffl, M. W. A new mathematical model for relative quantification in real-time RT-PCR. *Nucleic Acids Res* **29**, e45 (2001).

2 Deacon, R. M. & Rawlins, J. N. T-maze alternation in the rodent. *Nat Protoc* **1**, 7-12, doi:10.1038/nprot.2006.2 (2006).

3 Chomczynski, P.; Sacchi, N. Single-step method of RNA isolation by acid guanidinium thiocyanate-phenol-chloroform extraction. *Anal. Biochem.* **1987**, *162*, 156–159

Supplemental Table 1. Composition of the different diets

Ingredient	Control Diet (C)	High Fat Diet (HF)	Control Diet plus nopal (C+N)	High Fat Diet plus nopal (HFD+N)
Cornstarch	39.775	23.903	39.038	23.093
Casein (>85% protein)	20.000	24.000	19.417	24.417
Dextrinized cornstarch	13.200	10.267	13.200	10.267
Sucrose	10.000	7.778	10.000	7.778
Soybean oil	7.000	7.000	7.000	7.000
Fiber Cellulose	5.000	5.000	-	-
Mineral Mix (AIN-93G-MX)	3.500	3.500	3.500	3.500
Vitamin Mix (AIN-93-VX)	1.000	1.000	1.000	1.000
L-Cystine	0.300	0.300	0.300	0.300
Choline bitartrate (41.1% choline)	0.2500	0.2500	0.2500	0.2500
Tert-butylhydroquinone	0.0014	0.0014	0.0014	0.0014
Lard	-	17	-	17
Nopal	-	-	5	5

* The composition of the diets is given in g / 100g diet

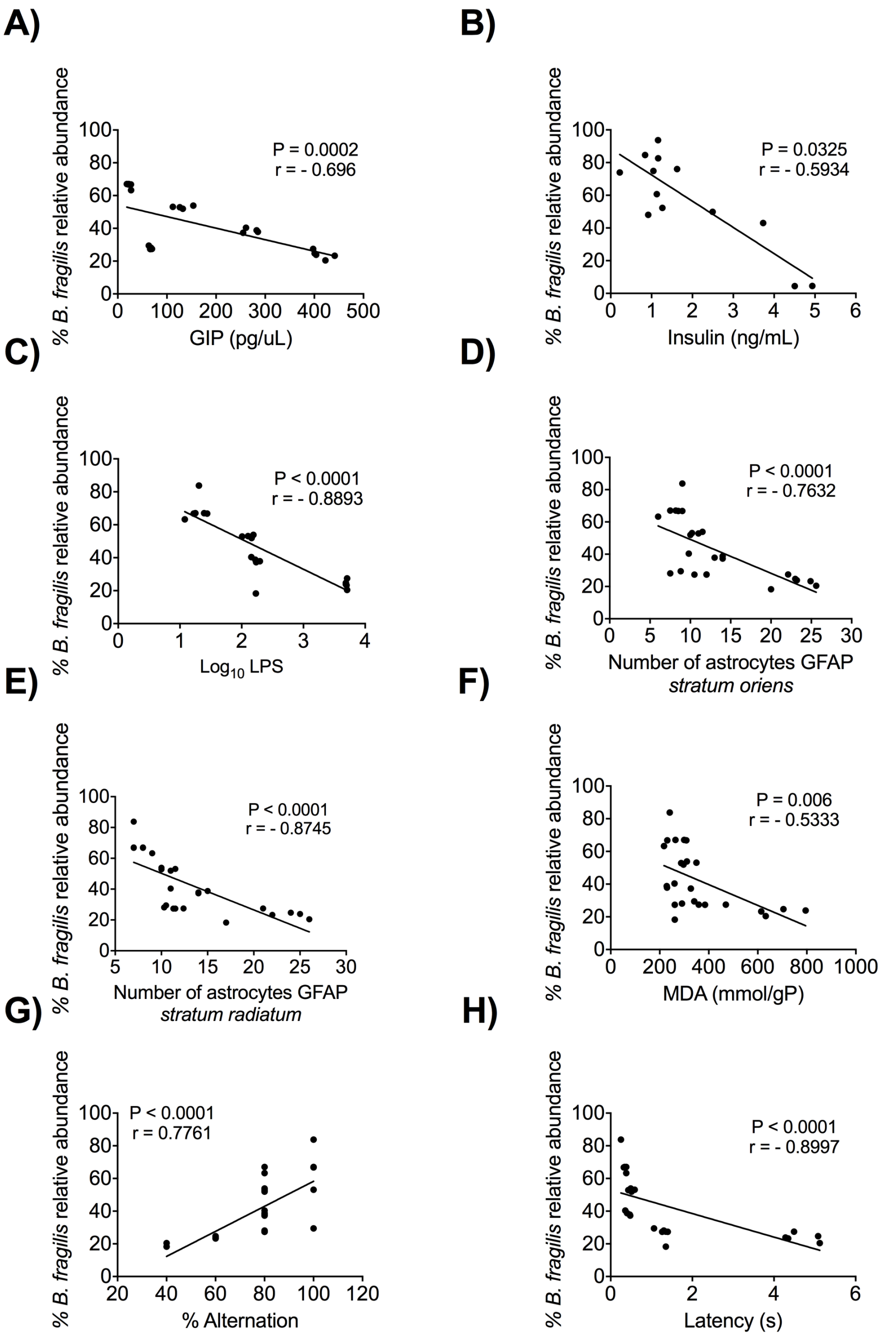

Supplementary Figure S1 Nopal consumption increases *B. fragilis* and reduces biochemical abnormalities and cognitive damage. Correlations between *B. fragilis* abundance and A) serum GIP, B) insulin, and C) lipopolysaccharide (LPS), D-E) GFAP in the stratum oriens and stratum radiatum of the ventral CA1hippocampus region, F) MDA in prefrontal cortex and G-H) % of alternation and latency in T-maze test. Data were analysed by Spearman correlation (n= 25).
